# Developing a digital intervention to combat fatphobia and anti-fat bias

**DOI:** 10.3389/fpsyt.2025.1569841

**Published:** 2025-05-13

**Authors:** Agatha A. Laboe, Elizabeth Sheil, Emma L. Jennings, Molly F. Steinhoff, Jake Goldberg, Kevin Sagat, Mahathi Gavuji, Katherine E. Schaumberg

**Affiliations:** ^1^ Department of Psychiatry, University of Wisconsin-Madison, Madison, WI, United States; ^2^ Department of Psychology, University of Texas at Austin, Austin, TX, United States; ^3^ Department of Psychology, Drexel University, Philadelphia, PA, United States; ^4^ Department of Psychological and Brain Science, Washington University in St. Louis, St. Louis, MO, United States

**Keywords:** eating disorders, digital intervention, college mental health, fatphobia, anti-fat bias, fear of weight gain, human-centered design

## Abstract

**Introduction:**

The Body Advocacy Movement (BAM) is an in-person, peer-led, cognitive-dissonance-based eating disorder (ED) prevention program that reduces fatphobia and anti-fat bias. Developing a digital adaptation of BAM has the potential to increase its accessibility and fill a critical gap in existing digital ED interventions, which to date have not specifically targeted anti-fat bias or fatphobia. This study applies a human-centered design approach to inform the development of a digital version of BAM.

**Methods:**

Semi-structured interviews were conducted with 31 participants, including 17 college students with elevated ED psychopathology and 14 past BAM participants. College students with elevated ED psychopathology shared experiences with fatphobia and anti-fat bias, how they use mental health technology, and thoughts on digitizing BAM. Past BAM participants shared experiences with BAM, how they use mental health technology, and thoughts on digitizing BAM. Interviews were analyzed using reflexive thematic analysis with a critical realist lens.

**Results:**

College students with elevated ED psychopathology described pervasive and harmful experiences of anti-fat bias and fatphobia, coupled with difficulties accessing action-oriented mental health support, underscoring a gap in care that a digital adaptation of BAM could address. Both groups expressed strong interest in a hybrid digital format that combines synchronous and asynchronous components for a balance of social connection and flexibility.

**Discussion:**

Findings suggest that a digital adaptation of BAM could address unmet needs in ED prevention by providing accessible, action-oriented content focused on reducing anti-fat bias and fatphobia. Incorporating synchronous social connection within a flexible, interactive framework may promote engagement and impact. A critical next step will involve designing and pilot testing this digital adaptation of BAM to evaluate its feasibility and effectiveness.

## 1 Introduction

Weight stigma refers to the societal devaluation of individuals based on body size or weight and is expressed through stereotypes (e.g., believing individuals at higher weights are lazy), prejudice (e.g., feelings of disgust toward individuals at higher weights) and discrimination (e.g., denying medical care to individuals at higher weights). It is prevalent among young adults and is linked to deleterious physical and mental health consequences (1). Burgeoning research suggests that in addition to weight-based stereotypes and discrimination, internalized weight stigma—the application of societal weight biases to oneself—is particularly harmful (2–4). Internalized weight stigma is associated with anxiety, depression, low self-esteem, body-image distress, and negative psychosocial outcomes across weight and gender groups (2–7). It is also linked to disordered eating behaviors, including dietary restraint, binge eating, and compensatory behaviors (2, 5). Furthermore, in one study of 8,504 young adults, those experiencing weight stigma and weight-related discrimination were 15.5% more likely to have an ED (8). Thus, preventing eating disorders (EDs) by addressing weight stigma—a rampant yet modifiable risk ED factor, with its own negative health effects—is paramount to promoting the well-being of young adults who are at high risk for developing an ED.

The Body Advocacy Movement (BAM) is a two-session, peer facilitated ED prevention program that employs cognitive dissonance and exposure-based strategies to combat two manifestations of weight stigma: fatphobia (i.e., the fear of becoming fat) and anti-fat bias (i.e., negative evaluations, stereotypes, or discrimination toward individuals perceived as fat), both of which are elevated among individuals with EDs (9–11). **Table 1** depicts the relationship between weight stigma, fatphobia, and anti-fat bias. To date, BAM has been implemented as a primary prevention program, delivered to a general college student population rather than specifically targeting individuals with elevated levels of anti-fat bias or fatphobia, although future work may focus on more targeted and/or high-risk samples.

**Table 1:** Weight stigma’s relation to fatphobia and anti-fat bias.

Term	Definition	Example	Relationship to Other Terms
Fatphobia	An individual’s fear of becoming fat and/or gaining weight.	Persistent unfounded fears that an individual will lose close relationships if they gain weight.	A specific, internal fear that can motivate or result from internalized weight stigma or anti-fat bias.
Anti-fat Bias	Negative attitudes, beliefs, or stereotypes about people perceived to be fat.	Assuming a fat person is unhealthy, lazy, or lacking self-control.	A cognitive and attitudinal expression of weight stigma; can be perpetuated by individuals or systems.
Internalized weight stigma	The adoption of negative societal beliefs about weight and applying them to oneself.	Feeling ashamed or unworthy because of one’s body size.	A self-directed outcome of exposure to weight stigma and/or anti-fat bias.
Weight stigma	The societal devaluation of individuals based on body size or weight. This stigma is embedded in cultural norms and systems and manifests through stereotypes, prejudice, and discrimination toward individuals in larger bodies.	Being denied medical care due to body size.	A broader, often systemic phenomenon; anti-fat bias, internalized weight stigma, and fatphobia are expressions or consequences of weight stigma.

BAM sessions, which are two hours long, are currently scheduled one week apart, and participants complete exposure-based exercises between meetings. In the first session, participants review terminology central to the intervention, including ‘fat’, ‘fatphobia’, and ‘anti-fat bias.’ They identify examples of fatphobia and anti-fat bias at three levels: (1) intrapersonal (i.e., within the self), (2) interpersonal (i.e., in interactions with others), and (3) institutional (i.e., within broader societal systems and organizations). Then, participants explore how fatphobia and anti-fat bias impact individuals of different body sizes. While some forms of discrimination specifically affect those at higher weights, concerns about weight gain can be present across the weight spectrum. After the session, participants complete an exposure-based ‘worst-case scenario’ exercise, in which they write about the most distressing possible consequences they associate with significant weight gain. At the beginning of the second session, participants are encouraged to share their ‘worst-case scenario’ reflections and identify strategies for challenging fatphobia. They then participate in a role-playing exercise, responding to and countering common messages rooted in fatphobia (e.g., ‘I really need to tone up’) and anti-fat bias (e.g., ‘I avoid being friends with fat people because they can’t be active with me’) produced by peer facilitators. Finally, the group brainstorms ways to combat anti-fat bias at the institutional level, and each participant shares one way they plan to act against institutional anti-fat bias. As a final reflection, participants write a response to their ‘worst-case scenario,’ incorporating insights from intervention discussions.

While BAM targets fatphobia and anti-fat bias, it is situated within a broader context in which these constructs are upheld by cultural norms, institutional practices, and systemic inequities. The intervention is not intended to place responsibility on individuals to ‘fix’ the consequences of societal oppression. Rather, BAM equips participants with tools to recognize and challenge fatphobia and anti-fat bias at multiple levels, including the institutional level, and is best understood as one component of a multi-level approach to stigma reduction that must also include policy change, public health advocacy, and structural reform.

Empirical research supports BAM’s effectiveness: it has been found to reduce fatphobia, anti-fat bias, and ED psychopathology (i.e., body dissatisfaction, binge eating, and excessive exercise) with small-to-moderate effects (12). While it was initially developed as an in-person intervention, digital implementation of BAM has the potential to increase its accessibility. Digital mental health interventions are widely used, effective, and perceived as helpful by young adults (13–18). Furthermore, multiple mobile health and internet-based interventions have proven effective at reducing ED symptoms (19, 20). However, existing digital interventions do not specifically address anti-fat bias or fatphobia, leaving a gap in ED prevention that could be addressed with a digital version of BAM.

A digital version of BAM has the potential to be widely disseminated, yet it will only be effective if its users are engaged and satisfied with its new format. Reviews of current mobile health technology indicate that dissatisfaction with the user experience, concerns about personalization and customizability, and a perceived disconnect between app functions and personal goals are primary reasons for participant disengagement from digital interventions, highlighting salient barriers to overcome with the digitization of BAM (13, 19, 21). A human-centered design approach, which centralizes the design of digital interventions on those who will be using them, may help circumvent these potential challenges (22).

Human-centered design is an iterative, multi-phase approach commonly used in the development of digital health tools to improve usability, acceptability, and real-world effectiveness (23). While several frameworks for human-centered design exist, the process typically encompasses six phases: (1) investigate, which includes understanding users’ needs, goals, and preferences, (2) ideate, during which ideas for designs are brainstormed, (3) prototype, when different iterations of a product are developed, (4) evaluate, during which prototype designs are finalized, (5) refine and develop, when usability testing is conducted and product design is optimized, and (6) validate, during which the product is tested (23). This study focuses on the investigate and ideate phases of human-centered design to inform the development of a digital adaptation of BAM.

In preparation for the study (ideate phase), we collaborated with eight BAM peer facilitators to generate five possible formats for the digital adaptation of BAM. These formats ranged from fully asynchronous to fully synchronous and included 1) an asynchronous, self-paced, individually-completed intervention that would be completed on a website, app, or other digital platform and would be a space to learn about anti-fat bias and fatphobia and challenge experiences of anti-fat bias and fatphobia; 2) the same asynchronous intervention, with the option to connect with a trained peer facilitator via a video call and/or messaging to discuss experiences completing the intervention; 3) the same asynchronous intervention, with the option to connect with others going through the intervention via a moderated online discussion board; 4) a mixed asynchronous and synchronous intervention, in which participants would complete activities on their own time on a website, app, or other digital platform, then would debrief on short video calls with a group and trained peer facilitator; and 5) a fully synchronous intervention in which BAM would be translated to a live, video call-based format. During semi-structured interviews, participants from both groups provided direct feedback on the five proposed adaptation ideas. Their insights helped evaluate the acceptability of and interest in each format, laying the foundation for future human-centered design phases.

In the next phase (investigate), we conducted semi-structured interviews with individuals from two key groups—college students with elevated ED psychopathology and past BAM participants. These groups were selected to capture complementary perspectives. College students with elevated ED psychopathology represent the intended users of the digital adaptation of BAM and provided insight into their experiences with anti-fat bias and fatphobia, as well as their perceptions of how a digital intervention could address these challenges. Their input was essential for identifying needs of the target population and anticipating potential barriers to engagement. Past BAM participants, who had firsthand experience of the in-person program, offered content-specific feedback on which elements should be retained, adapted, and reimagined in a digital format, helping to ensure the relevance and integrity of BAM’s core components.

Overall, this study demonstrates how early-phase human-centered design can be used to inform the development of a digital adaptation of BAM, an ED prevention program, that is both evidence-based and responsive to the needs of intended users. By integrating perspectives from both target users and individuals familiar with intervention content, findings will guide design decisions to enhance user engagement and ultimately, clinical impact.

## 2 Materials and methods

### 2.1 Participants and recruitment

College students with elevated ED psychopathology were recruited through social media, as well as direct outreach based on participation in prior studies conducted by the study team. Before enrolling in the study, their eligibility was assessed via an online self-report eligibility screen. They were eligible if they were at least 18 years old, were enrolled at any college or university at any level of study, were English-speaking, and had a probable ED or were at high risk for an ED based on results from the Stanford-Washington University ED Screen (SWED; 24). Of the 23 people who completed the eligibility screen, four were ineligible based on results from the SWED (i.e., were considered low risk based on their responses) and were notified of their ineligibility for the study. Two were lost to follow-up and the remaining 17 participated in the study.

Past BAM participants were originally recruited to complete BAM from the general Madison, Wisconsin area through (1) flyers at businesses (e.g., fitness centers, coffee shops), (2) social media, (3) direct, peer-to-peer recruitment by peer facilitators, and (4) university clubs and organizations. Eligibility criteria for BAM include being English-speaking and between the ages of 18 and 30 years old. Past BAM participants were eligible for the current study if they completed both sessions of an in-person BAM workshop in in February or March of 2024. They were informed of the study at the end of the second session and could opt in to being contacted about participating by supplying their contact information on a study sign-up sheet. Of the 19 individuals who completed a BAM workshop in February or March of 2024, 15 supplied their contact information, and 14 ended up completing the study.

### 2.2 Procedure

#### 2.2.1 College students with elevated ED psychopathology

**Figure 1:**
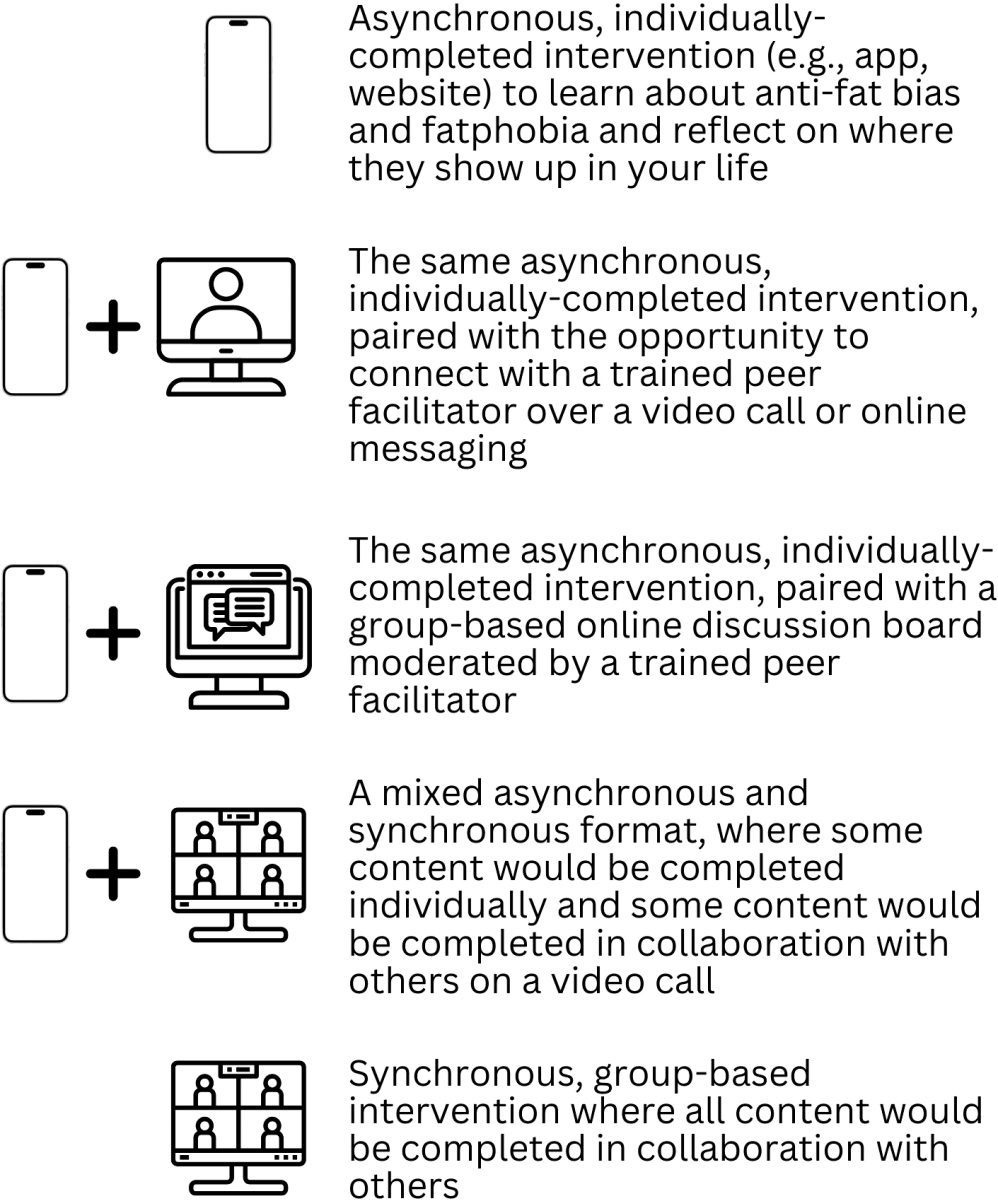
Digital BAM adaptation ideas.

College students with elevated ED psychopathology first provided consent online. Then, they completed an online questionnaire, including the measures described below. Upon completion of the questionnaire, participants completed a one-on-one, video-call based, audio-recorded semi-structured interview with a study team member during which they shared their experiences with fatphobia and anti-fat bias, how they use technology to support their mental health, and their thoughts on the five different BAM digital adaptations ideated by the study team (see **Figure 1**). The interview was expected to take 60 minutes (range = 25–75 minutes). Participants were compensated $25 upon completion of these study activities.

#### 2.2.2 Past BAM participants

After providing informed consent online, past BAM participants took part in a semi-structured interview that was one-on-one, video-call based, and audio-recorded. During the interview, they reflected on their experiences with BAM, how they use technology to support their mental health, and their thoughts on adapting BAM to a digital format. Participants shared their initial ideas for digitizing BAM and then provided feedback on five digital BAM adaptations ideated by the study team (see **Figure 1**). The interview was expected to take 60 minutes (range = 30–65 minutes).

Notably, most past BAM participants (n=13/14) were enrolled in a separate study testing the effectiveness of the in-person BAM workshop. For this separate study, they completed online questionnaires at baseline (i.e., prior to completing the workshop), immediately after completing the workshop, and 8 weeks after completing the workshop. During the informed consent process, participants agreed to allow data collected during their participation in this separate study to be used in the present study. For the present study, their baseline data for the measures described below was included. The one past BAM participant who did not participate in the separate BAM study completed the online questionnaire, including the measures described below, upon completion of their interview. Participants were compensated $25 for their participation in this study, which included both the interview and the online questionnaire.

This study received a Not Human Subjects Research exemption from ethical approval and written informed consent. The study was conducted in accordance with local legislation and institutional requirements, and all participants indicated their agreement online prior to continuing with the study after the study was described.

### 2.3 Measures

#### 2.3.1 Descriptive data

Demographic information, including gender, age, race, and sexual orientation, was collected from both groups. Descriptive data (see below) was also collected from both groups to contextualize qualitative findings and explore patterns that may inform future research.

The ED100K Plus was administered to gauge levels of ED psychopathology. The ED100k Plus combines selected items from the ED100k (25; 90 items), the Eating Disorder Diagnostic Scale (26; 10 items), the Eating Disorder Examination-Questionnaire (27; 40 items), and the Dieting and Weight History Questionnaire (28; 10 items), along with 54 additional questions to clarify all criteria needed to assess current and historical diagnoses of DSM-5 EDs. Questions were administered logically with minimal responses necessary; thus, portions of the assessment detailing certain ED experiences were skipped if screening items related to these experiences were not endorsed. Average time to completion for the ED100k Plus was 17.4 minutes. This self-report questionnaire uses DSM-5 criteria to assess both current and history of: anorexia nervosa, atypical anorexia nervosa, bulimia nervosa, subclinical bulimia nervosa, binge eating disorder, subclinical binge eating disorder, and purging disorder.

The Goldfarb Fear of Fat Scale (GFFS) was used to measure fatphobia (29). The GFFS is a 10-item measure designed to evaluate an individual’s thoughts and fears related to becoming fat. Participants rate each item on a 4-point Likert scale ranging from 1 (“Very untrue”) to 4 (“Very true”), with total scores ranging from 10 (indicating no fear of fat) to 40 (indicating extreme fear of fat). The GFFS has demonstrated strong psychometric properties and test-retest reliability across both clinical and non-clinical populations (30). Cronbach’s alpha for the GFFS with the present sample was 0.83 for college students with elevated ED psychopathology and 0.89 for past BAM participants, demonstrating good internal consistency.

The Eating Psychopathology Symptoms Inventory-Negative Attitudes Towards Obesity (EPSI-NATO) subscale is one of eight subscales in the EPSI that was used to measure levels of anti-fat bias in this study (31). The EPSI-NATO is a 5-item subscale that assess feelings towards individuals in larger bodies. Participants rate each item on a 5-point Likert scale from 0 (“Never”) to 4 (“Very Often”) and the responses are then summed to create a total score. The EPSI has been validated in both clinical and college samples (32). Cronbach’s alpha for the EPSI NATO subscale with the present sample was 0.90 for college students with elevated ED psychopathology and 0.90 for past BAM participants, demonstrating excellent internal consistency.

Finally, during the semi-structured interview, participants shared their interest in participating in five different BAM digital adaptations ideated by the study team using a whole-number scale from 1 (not at all interested) to 5 (very interested). These adaptation ideas were presented in a counterbalanced order and are described above and depicted in **Figure 1**. The purpose of these ratings was not to draw definitive conclusions about preference strength, but rather to complement qualitative data with a basic snapshot of comparative interest across five design ideas.

#### 2.3.2 Qualitative data

Semi-structured interviews with college students with elevated ED psychopathology were conducted to answer the research questions: How do college students with elevated ED psychopathology experience anti-fat bias and fatphobia, and how might a digital intervention address these experiences? Interviews began by getting a sense of current struggles with body image and food (i.e., “I’m curious to hear about your struggles with body image and food. What does this look like for you right now?”). From there, definitions of fatphobia and anti-fat bias were reviewed and personal experiences and observations of others’ experiences with fatphobia and anti-fat bias were assessed (e.g., “How have experiences of fatphobia affected your mental health or well-being?” “Have you ever noticed fatphobia in others?” “Have you ever been on the receiving end of anti-fat bias?”). Participants were then asked about their prior use of technology to support their mental health (e.g., “Are you currently using technology to support your mental health or well-being?”). Finally, they were informed that the research team was developing a digital intervention to address anti-fat bias and fatphobia. Participants were presented with five preliminary adaptation ideas, one at a time, then provided their reactions and suggestions (e.g., “What are your initial reactions to this idea?”).

Semi-structured interviews with past BAM participants were conducted to answer the research question: What aspects of BAM should be retained or modified to effectively adapt the program to a digital format? Interviews began by reviewing an outline of BAM and assessing feedback on BAM (e.g., “How did you feel about the time commitment?” “How relevant did you find BAM to you, personally? Can you give an example?” “What information/session/activity stood out to you the most? Why)?. Participants were then asked about their use of technology to support their mental health (e.g., “Have you ever used technology to support your mental health or well-being?”). Finally, they were informed that the research team was developing a digital version of BAM and were presented with the five adaptation ideas, one at a time, for which they provided their reactions and suggestions (e.g., “What are your initial reactions to this idea?”).

### 2.4 Analytic strategy

#### 2.4.1 Descriptive analysis

Means and standard deviations (SDs) for the quantitative measures were calculated for each group to provide descriptive context about the sample. Independent samples t-tests were conducted to examine potential group differences on psychological constructs of interest. Mann Whitney U-tests were conducted to examine potential group differences on ratings of digital adaptation ideas. While these analyses were not intended to formally test hypotheses and were underpowered, t values, U values and p-values are reported for transparency, and effect sizes are included to support interpretation and contextualize observed patterns.

#### 2.4.2 Qualitative analysis

Team-based, reflexive thematic analysis was used to develop themes from participants’ experiences (33). Grounded in a critical realist ontology, our analysis acknowledged the interplay between participants’ subjective experiences and the broader social, cultural, and structural influences (e.g., weight stigma, cultural ideals of appearance, disparities in access mental health treatment) shaping these experiences. We adopted an inductive approach, meaning our analysis was driven directly by the data rather than prior theory or research. Within a critical realist framework, this inductive process allowed us to identify and contextualize how subjective experiences were shaped by external forces. Reflexivity also played a central role in this process, as described further below.

Prior to analysis, interviews were transcribed manually based on audio-recordings. During transcription, identifying information (e.g., school name) was replaced with a non-identifying description (e.g., [School Name]) and participants were assigned participant pseudonyms which are used below when quoting individual interviews. Coding and analysis were led by the first author using Braun and Clarke’s (33) approach to reflexive thematic analysis. The same coding and analytic process, detailed below, was completed separately for past BAM participants and college students with elevated ED psychopathology.

First, members of the coding team (A.A.L., M.F.S., E.S., E.L.J., J.G., K.S.) individually read all transcripts to assess the size and adequacy of the dataset in relation to information power (i.e., breadth and depth of the data given the study’s aim and approach; 34). Review of the data indicated sufficient richness to complete analysis, so the team moved forward with analysis instead of collecting more data. To develop the coding guides, each member of the coding team inductively generated initial codes. Then, the coding team met to refine codes and create an initial coding guide, which was used for test-coding of 12 transcripts (each member of the coding team test-coded two transcripts). After test-coding, the coding team met again to finalize the coding guide, which comprised semantic codes (i.e., codes that capture surface-level meaning of the data). From there, each member of the coding team coded 8–14 transcripts total, across groups, using *Dedoose*, version 9.2.22. Each transcript was coded twice, by two different coders. Coding discrepancies were identified and discussed in coding pairs, fostering reflexivity and deeper insight into the data. For initial theme generation, the coding team collaboratively clustered codes, drawing on their familiarization with and understanding of the data. Once initial themes were generated, the first author went back to the dataset to confirm the themes accurately represented the data and further revised the themes, which the team reviewed.

At the core of reflexive thematic analysis is the understanding that research is inherently subjective, necessitating reflexivity throughout the research process (33). Within the present analysis, reflexivity was both individual and collaborative. All interviews were conducted by the first author, who identifies as a White, heterosexual, smaller-bodied woman. Her lived experience with anti-fat bias, fatphobia, and an ED provided her with a personal connection to the topics discussed, fostering empathy and rapport with participants. Hearing participants’ struggles firsthand underscored, for her, the need to increase accessibility of BAM, a sentiment she discussed frequently with M.G. and K.E.S.

Before analyzing transcripts, the coding team engaged in bracketing exercises to reflect on their positionalities and preconceived ideas about digital interventions for eating disorders. This process aimed to increase awareness of potential assumptions that might shape data interpretation. Some team member assumptions included: (a) engagement will be a challenge with digitizing BAM; (b) experiences of anti-fat bias and fatphobia are common; and (c) a digital version of BAM would increase accessibility. The coding team brought disciplinary expertise in psychology and medicine, and multiple members have lived experience of EDs and/or working with patients with EDs. The team also reflected certain demographic characteristics: White (6/6), heterosexual (3/6), woman-identifying (4/6), smaller-bodied (6/6), and holding Bachelor’s degrees as their highest level of education (3/6). Recognizing that these identities were similar in some instances, and different in others, to identities of the participants, the team prioritized regular reflexive discussions to critically examine how their positionalities might influence the analysis. These collaborative discussions shaped the analysis by prompting the team to question initial interpretations, ensure that findings were grounded in the data, and consider how both shared and differing perspectives informed the themes identified. For example, frequent discussions of anti-fat bias and accessibility led the team to interrogate whether they were unintentionally prioritizing themes aligned with their professional goals over less familiar participant perspectives. This iterative reflexive process ensured the analysis remained attuned to participants’ experiences.

## 3 Results

### 3.1 Descriptive statistics

A total of 31 participants were enrolled, including 17 college students with elevated ED psychopathology and 14 past BAM participants. Grounded in information power, these sample sizes were determined to be sufficient for a comprehensive exploration of themes and meaningful group comparisons (34). Participants were predominantly woman-identifying (94.1% college ED; 71.4% past BAM) and White (76.5% college ED; 57.1% past BAM), with a mean age of 22.6 years for college students with elevated ED psychopathology and 23.4 years for past BAM participants. The mean age of both groups reflects the inclusion of graduate students. Most participants fell into a Mid-Range Body Mass Index (BMI) category of 18.5-24.9 (58.8% college ED; 71.4% past BAM), based on self-reported height and weight. See **Table 2** for full details on study participant demographics.

**Table 2:** Sample demographics.

Variable	College ED (N=17)	Past BAM (N=14)
Gender Identity: N (%)		
Man	1 (5.88%)	2 (14.29%)
Nonbinary	0 (0.00%)	2 (14.29%)
Woman	16 (94.12%)	10 (71.43%)
Gender Identity – Transgender/Cisgender: (N%)		
Cisgender	15 (88.24%)	12 (85.71%)
Transgender	1 (5.88%)	2 (14.29%)
Unsure	1 (5.88%)	0 (0.00%)
Race: N (%)		
Asian	3 (17.65%)	5 (35.71%)
Black	1 (5.88%)	0 (0.00%)
Pacific Islander	0 (0.00%)	1 (7.14%)
White	13 (76.47%)	8 (57.14%)
Sexual Identity: N (%)		
Asexual	2 (11.76%)	1 (7.14%)
Bisexual/Bi+/Pansexual	3 (17.65%)	6 (42.86%)
Heterosexual	11 (64.71%)	7 (50.00%)
Lesbian/Gay	1 (5.88%)	0 (0.00%)
Age: Mean (SD) [Range]	22.59 (2.76) [19-28]	23.43 (3.65) [19-29]
BMI: Mean (SD) [Range]	23.17 (3.94) [17.9-31.2]	23.44 (3.46) [18.7-31]
Eating Disorder Diagnosis*		
Anorexia Nervosa History	1 (7.1%)	5 (29.4%)
Current Anorexia Nervosa	0 (0%)	0 (0%)
Atypical Anorexia Nervosa History	2 (14.3%)	6 (35.3%)
Current Atypical Anorexia Nervosa	1 (7.1%)	2 (11.8%)
Bulimia Nervosa History	0 (0%)	1 (5.9%)
Current Bulimia Nervosa	0 (0%)	1 (5.9%)
Binge Eating Disorder History	0 (0%)	1 (5.9%)

*In line with the DSM-5, participants could only have one current eating disorder diagnosis at a time, but could report multiple historical diagnoses.

Regarding ED psychopathology, based on the ED100k Plus, a greater proportion of college students with elevated ED psychopathology reported a lifetime ED diagnosis (n = 13, 76.5%) compared to past BAM participants (n = 6, 42.9%). This difference aligns with sample eligibility criteria: college students with elevated ED psychopathology were required to screen as ‘at risk’ or have a probable ED based on the SWED, whereas this criterion did not apply to past BAM participants. Notably, not all college students reported a lifetime ED diagnosis, as being classified as ‘at risk’ on the SWED was sufficient for study enrollment. See **Table 2** for a complete breakdown of ED diagnoses.

To further characterize the sample, exploratory, descriptive group comparisons were conducted for psychological constructs. These analyses were descriptive in nature and not intended to test formal hypotheses but rather to provide preliminary insights into group differences that may inform future research. Effect sizes are reported to provide context for interpreting these preliminary patterns. College students with elevated ED psychopathology scored slightly higher on the GFFS (M = 12.82, SD = 5.55) compared to past BAM participants (M = 10.14, SD = 7.36), *t* = 1.12, *p* = 0.272, with a medium positive effect size (d = 0.41). On the EPSI NATO, past BAM participants scored higher (M = 5.29, SD = 5.11) compared to college students (M = 4.53, SD = 4.21), *t* = -0.45, *p* = 0.656, with a small negative effect size (d = -0.16). See **Table 3** for full details.

**Table 3:** Fears of fat and negative attitudes toward obesity across groups.

Variable	College ED – Mean (SD)	Past BAM – Mean (SD)	t value	p value	Cohen’s d
GFFS	12.82 (5.55)	10.14 (7.36)	1.12	0.272	0.41
EPSI NATO	4.53 (4.21)	5.29 (5.11)	-0.45	0.656	-0.16

### 3.2 Digital adaptation idea preferences

Participants rated their interest in five digital adaptation ideas (see **Figure 1**) for BAM using a Likert scale (ranging from 1 = not at all interested to 5 = very interested), and comparisons were made between college students with elevated ED psychopathology and past BAM participants. Descriptively, the mix of asynchronous and synchronous format received the highest average ratings from both groups, with the mean score for college students with elevated ED psychopathology being 3.94 (SD = 0.75) and the mean score for past BAM participants being 3.57 (SD = 1.34). In contrast, the fully asynchronous format received the lowest average ratings from both groups, with the mean score for college students with elevated ED psychopathology being 2.94 (SD = 1.39) and the mean score for past BAM participants being 2.29 (SD = 1.27). A statistically significant difference between groups emerged for the asynchronous plus peer facilitator format (*U* = 46.5, *p* = .003). College students with elevated ED psychopathology rated this format significantly higher (Median = 4, IQR = 1) than past BAM participants (Median = 3, IQR = 0.75), demonstrating a large effect size (*r* = 0.539), where *r* represents the rank-biserial correlation derived from the z-approximation of the Mann-Whitney U test. See **Table 4** for full details of the ratings and comparisons across all formats.

**Table 4:** Digital adaptation of BAM ratings.

Digital Adaptation Idea	College ED – Mean (SD)	Past BAM – Mean (SD)	College ED - Median (IQR)	Past BAM – Median (IQR)	U value	p value	Effect size (r)
Fully Asynchronous	2.94 (1.39)	2.29 (1.27)	3 (2)	2 (2.75)	88.0	0.212	0.224
Asynchronous + Peer Facilitator	3.76 (0.97)	2.57 (0.94)	4 (1)	3 (0.75)	46.5	0.003	0.539
Asynchronous + Online Discussion Board	3.29 (1.21)	3.07 (1.33)	3 (2)	3 (2)	108.0	0.669	0.081
Asynchronous + Synchronous Meetings	3.94 (0.75)	3.57 (1.34)	4 (1)	4 (2.5)	107.5	0.646	0.086
Fully Synchronous	3.41 (1.18)	3.57 (1.28)	4 (1)	4 (1.75)	129.5	0.682	0.077

### 3.3 Qualitative results: College students with elevated ED psychopathology

**Figure 2:**
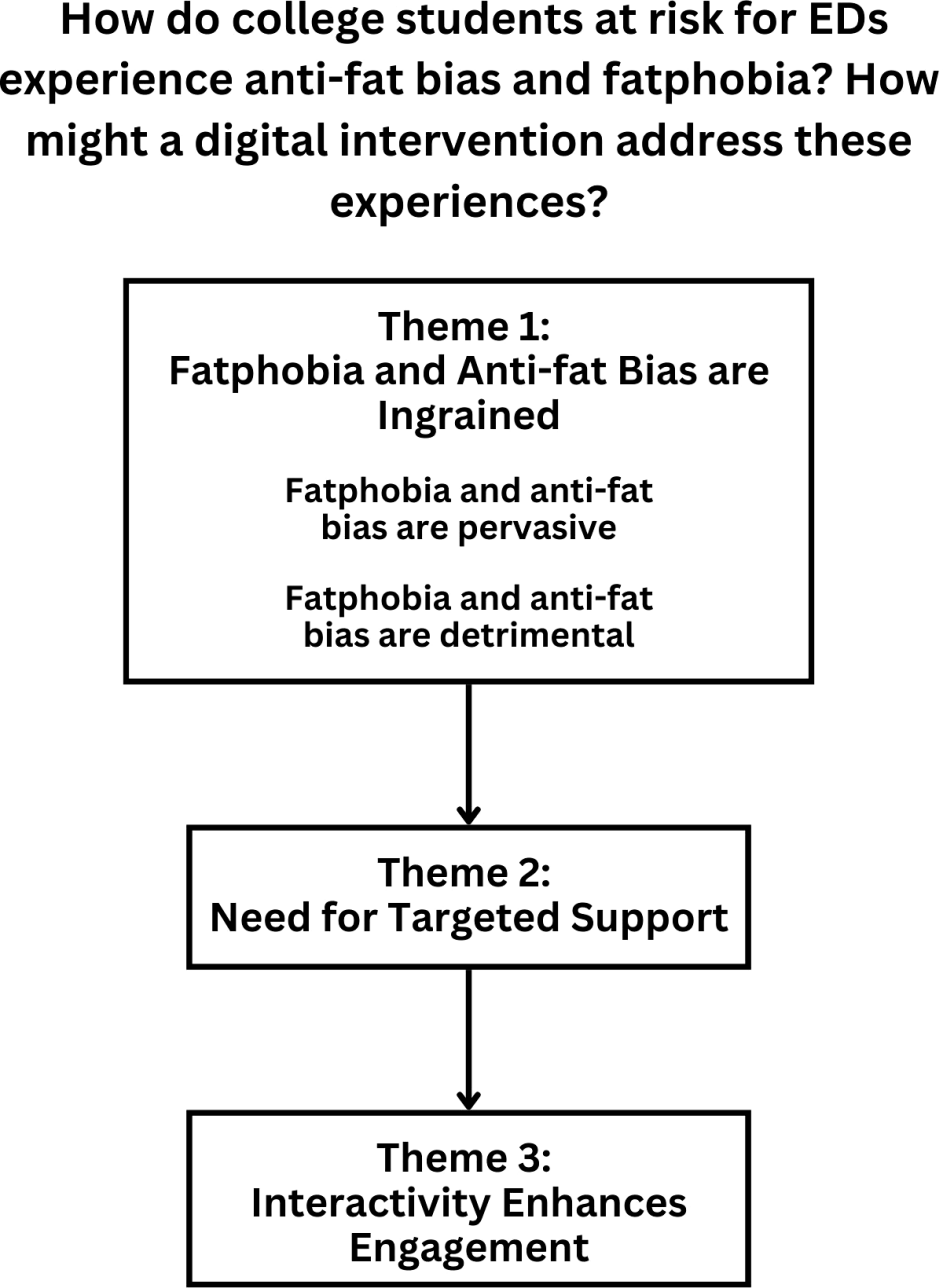
Need for a digital adaptation of BAM.

The following analysis explores the themes identified through semi-structured interviews with college students with elevated ED psychopathology. **Figure 2** depicts these themes in relationship with one another. Ultimately, this analysis answers the research questions: How do college students at risk for EDs experience anti-fat bias and fatphobia, and how might a digital intervention address these experiences? We use participant pseudonyms when sharing quotes and include a participant quote when presenting each theme. **Table 5** provides demographic and descriptive information for college students at risk for EDs for contextualization.

**Table 5:** College ED participant pseudonyms, demographic information, and ED history.

Pseudonym	Race/Ethnicity	Gender	BMI^a^	ED History
Stephanie	White	Woman	Lower BMI	Yes
Lauren	White	Woman	Higher BMI	No
Alicia	Asian	Woman	Mid-Range BMI	Yes
Scott	White	Man	Mid-Range BMI	Yes
Anna	White	Woman	Mid-Range BMI	Yes
Jessie	White	Woman	Mid-Range BMI	No
Sarah	White	Woman	Higher BMI	No
Bridget	Asian	Woman	Mid-Range BMI	No
Annie	White	Woman	Mid-Range BMI	Yes
Maria	White	Woman	Higher BMI	Yes
Caitlin	White	Woman	Mid-Range BMI	Yes
Tatiana	White	Woman	Higher BMI	Yes
Claire	Black	Woman	Higher BMI	Yes
Irene	White	Woman	Mid-Range BMI	Yes
Coco	Asian	Woman	Mid-Range BMI	Yes
Courtney	White	Woman	Lower BMI	Yes
Alexis	White	Woman	Higher BMI	Yes

a Lower BMI = BMI<18.5, Mid-Range BMI= 18.5-24.9, Higher BMI=BMI>25, determined based on self-reported height and weight.

#### 3.3.1 Theme 1: Fatphobia and anti-fat bias are ingrained: “knee-jerk reactions” (Irene)

Participants described in detail the insidious nature of fatphobia and anti-fat bias in their daily lives, highlighting their presence across intrapersonal, interpersonal, and institutional domains. They reflected on how these biases affected them personally and shaped their interactions with others. Notably, *all* participants shared experiences of fatphobia and anti-fat bias (as targets of this bias or as individuals who perpetuated it).

##### 3.3.1.1 Subtheme 1: Fatphobia and anti-fat bias are pervasive: “always coming up in conversation” (Bridget)

Participants identified several specific contexts in which they frequently experienced fatphobia and anti-fat bias, such as “eating out,” “dressing up,” “exercising at the gym,” “at the doctor,” and “in conversations with friends and family.” Bridget shared, “[Fatphobia] comes up in conversations that, ‘Ah, I’m getting fat … I’m putting on weight, so I feel like I should start going to the gym.’ Sometimes … friends say that to me. Family-wise … my mom’s always overly concerned with her weight and body image.” Tatiana expressed, “My brother and I have both been … called obese … at the doctor’s office … [The doctors] show you … a chart and … a bunch of … normal curves and they’re like, ‘This is a healthy person at your weight. This is a really active person at your height and weight. This is like a less active person. And this is you.’ They find somewhere way off the curves and they’re like, ‘Yep, this looks really bad. You’re so obese.’” Participants also discussed anti-fat bias they held themselves. For example, Irene admitted, “I think my initial reaction to seeing a fat person or talking to them is like, ‘You eat everything. You have no self-control. You can’t make yourself do anything.’ And then it’s like obviously resetting my brain and being like, that’s not a thing.” Participant accounts illustrated the pervasive and multifaceted nature of fatphobia and anti-fat bias, showing how they manifest not only internally but also interpersonally and institutionally.

##### 3.3.1.2 Subtheme 2: Fatphobia and anti-fat bias are detrimental: “driving forces behind my eating and body image struggles” (Maria)

Participants frequently reflected on the profound impact experiences of anti-fat bias and fatphobia had on their mental health, body image, and eating behaviors. Sarah reflected on how anti-fat bias affected her sense of self-worth, explaining, “I’ve gone through periods of time where I felt like my parents aren’t as proud of me as they would be if I was thin.” Participants also discussed how fatphobia and anti-fat bias contributed to the development or exacerbation of their eating disorders. For example, Alexis described the emotional toll of experiencing anti-fat bias and how it fueled her disordered eating behaviors: “There was all the shame, embarrassment, guilt for the body that I had that I couldn’t make it different … I couldn’t deal with it anymore … I couldn’t deal with … how I felt after people … were making comments [rooted in anti-fat bias] … That anger and the lack of control is what fueled … the eating disorder.”

The emotional burden of fatphobia extended beyond disordered eating, with participants describing its pervasive influence on their mental health. For example, Anna shared, “[Fatphobia] is one of the harder things I’ve gone through in the past … For whatever reason, it really consumes my brain … It makes me super anxious. I feel like it’s contributed to a lot of my sadness. So yeah, it’s … definitely been … a big struggle for me. It started a little bit after college started and … I’m gonna be a senior—so in a way, it’s been a big part of my journey [in college].”

Family dynamics were another area where participants experienced the detrimental effects of fatphobia. Several participants reflected on how comments and attitudes from family members, particularly mothers, shaped their body image and anxieties. Maria explained: “I think [my mom’s fatphobia] definitely … made me more aware of my body growing up which probably … in turn contributed to me having these anxieties about my body and stuff like that. And hearing that growing up, I think it’s tough especially when you’re … a little girl and you … see your mom and you think your mom is so pretty and then … all she says is like ‘I don’t think I’m pretty,’ so it’s like well then I don’t think I’m pretty because I look like you. And that’s kind of really hard.” The emotional and behavioral experiences participants described highlight the detrimental effects of anti-fat bias and fatphobia.

#### 3.3.2 Theme 2: Need for targeted support: “I’m looking for a solution” (Scott)

Participants emphasized a need for specific, focused support for their struggles with eating and body image concerns, as well as related issues of fatphobia and anti-fat bias. Many participants described negative experiences with therapy, particularly due to working with therapists who were not ED-informed and a lack of direction in therapy sessions.

Maria reflected on her experience working with a therapist who lacked training in EDs, sharing, “[My therapist] didn’t really know what to do [when I brought up my body image struggles], I think. I would tell her about … what I was feeling and she wouldn’t necessarily really … validate me at all … So when that was going on, I just kind of … shut down from the process because I was like, well, I’m not getting what I want out of this.” Similarly, Alexis explained how working with a therapist unfamiliar with EDs could feel invalidating and even be harmful. She shared, “I’m working with this therapist on other stuff and … occasionally … the ED does come up because, I mean, it’s part of my mental health … and not that they’re doing it intentionally, but it’s just they don’t know how to talk about it. So there can be … potentially triggering things that are said or … misunderstandings about how an ED works so … I would say that’s not a helpful thing.” These experiences reflect a common frustration among participants who felt their therapists were not equipped to provide the specialized care they needed for body image and eating struggles.

Participants also emphasized that a lack of structure in therapy sessions often made therapy feel unproductive. Irene described her experience bluntly: “Counseling is … a waste of time … I just feel like we talk and talk and talk and accomplish nothing.” Scott echoed this frustration, highlighting the need for action-oriented approaches, sharing, “Too much listening [has been unhelpful in therapy] because it was just kind of me saying, ‘Here’s what’s going on. Here’s the problem. This is probably why it happens.’ Too much listening and not enough … questions or action plans [is unhelpful].” Even in group therapy settings, participants expressed a need for more structure and focus on practical skills. Stephanie shared, “I feel like maybe we needed to … have more organization. How these [group therapy was] conducted was just … very free for all to talk about whatever … It’s better if they were … focusing on some coping skills besides ‘Let’s just talk.’”

Overall, participants’ accounts highlighted significant gaps in therapy experiences, with many feeling that therapy fell short in addressing their eating and body image concerns. Whether due to a lack of ED-specific expertise or the absence of clear, goal-directed approaches, these findings underscore the need for targeted support for individuals struggling with these issues.

#### 3.3.3 Theme 3: Interactivity enhances engagement: “the more interactive, the better” (Annie)

Participants emphasized the importance of interactivity in the proposed digital adaptation of BAM, describing it as a key factor in maintaining engagement and promoting meaningful learning. Feedback centered on the value of both interactive activities and opportunities to interact with others.

Participants highlighted the need for engaging, interactive content that went beyond passive learning. Annie shared, “Quizzes would keep me engaged.” Maria suggested, “If you made … interactive content that might honestly be better than having someone just … presenting something. I feel like you could get more out of it … I’m thinking matching activities or … drag and drop.”

In contrast, participants expressed that less interactive formats, such as discussion boards, felt uninspiring and lacked engagement. Irene explained, “I don’t think [the asynchronous + group discussion board adaptation] would go too well … It kind of just makes it feel like a homework assignment that you’re doing to get it over with.” Alicia noted that interactivity was critical to ensuring sustained engagement across a diverse audience, sharing, “If there are some activities … where they can engage with the intervention itself, instead of just, only reading about the information, I think that would be good. I feel like only people who have an interest would continue reading and then those who might not would probably … have no inclination to continue further.”

Participants also emphasized the importance of social interaction in enhancing engagement. For example, Coco noted, “[The fully asynchronous version] is … not bad, but it might not differentiate itself from other things … [The human connection] would make … everything stick a bit better and be more applicable.” Similarly, Scott expressed that he would be “more inclined to do [the asynchronous + group discussion board adaptation] than the purely asynchronous one because it would be nice to have that community aspect. It would be nice to have the external support.” The opportunity to connect with a peer facilitator was also highlighted as a key motivator for participation. Alexis reflected, “I think that having at least the opportunity [to connect with a peer facilitator] would probably pull a lot more people to utilize it … I just think that … the opportunity to work one-on-one with someone is comforting.” Others emphasized the value of group-based interactions. For instance, Anna shared, “I would prefer the [fully synchronous version] just because I think that group aspect is really helpful to grow and learn from.”

Overall, participants emphasized that interactivity—whether through interactive activities or interactions with others—would be critical to fostering engagement in a digital adaptation of BAM.

### 3.4 Qualitative results: Past BAM participants

**Figure 3:**
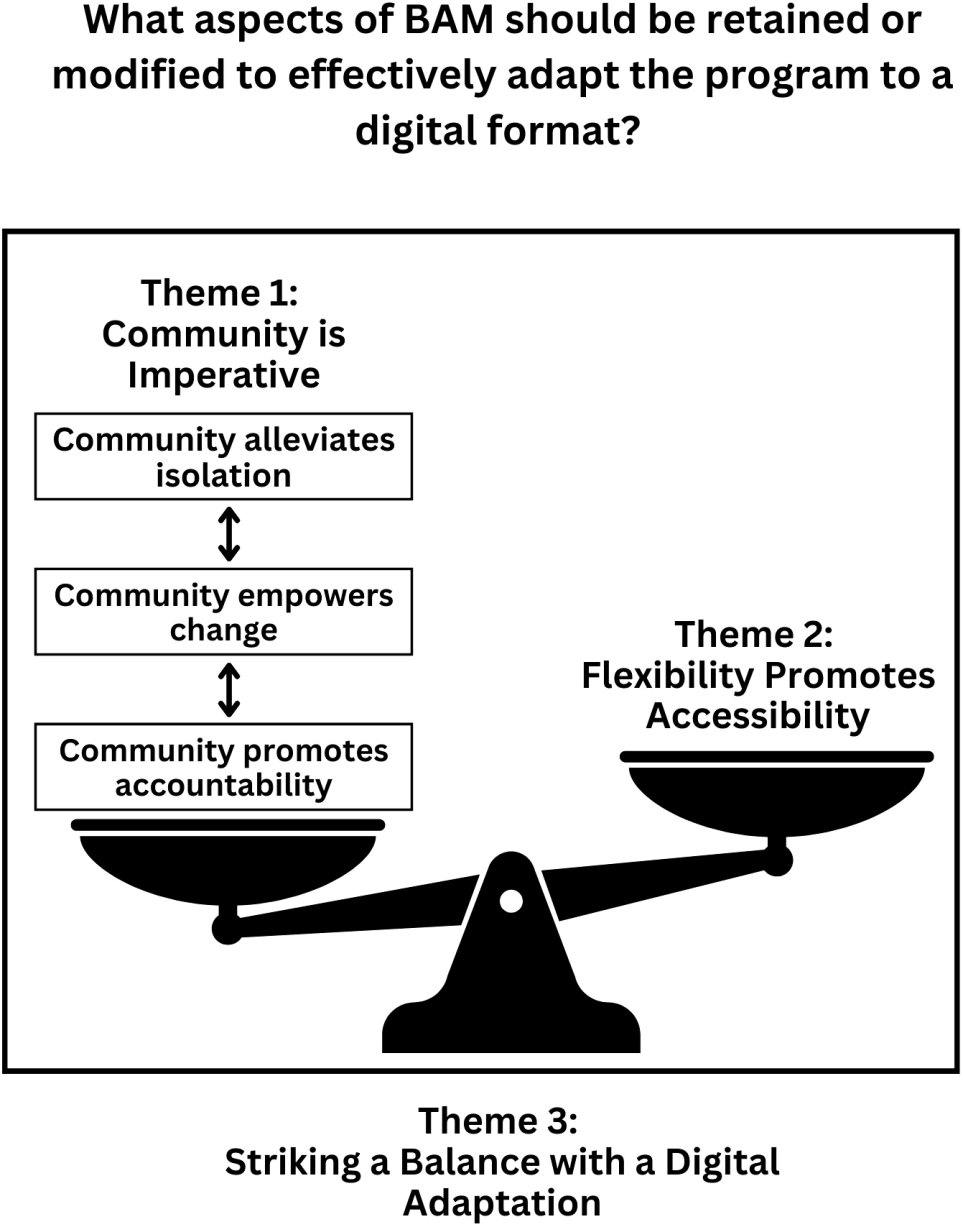
Adapting BAM to a digital format.

The following analysis explores the themes in past BAM participants’ responses during the semi-structured interviews. **Figure 3** depicts these themes in relationship with one another, highlighting past BAM participants’ perspectives on a digital adaptation of BAM. Ultimately, this analysis answers the research question: What aspects of BAM should be retained and modified to effectively adapt the program to a digital format? We use participant pseudonyms when sharing quotes and include a participant quote when presenting each theme. **Table 6** provides demographic and descriptive information for past BAM participants for contextualization.

**Table 6:** Past BAM participant pseudonyms, demographic information, and ED history.

Pseudonym	Race/Ethnicity	Gender	BMI^a^	ED History
Isabel	Asian	Woman	Mid-Range BMI	No
Helen	White	Woman	Mid-Range BMI	No
Katie	Asian	Woman	Higher BMI	Yes
Joey	White	Man	Higher BMI	No
Alex	Asian	Nonbinary	Mid-Range BMI	No
Janet	Hispanic/Latinx	Woman	Mid-Range BMI	No
Marcia	White	Woman	Lower BMI	Yes
Rachel	White	Woman	Higher BMI	Yes
Ellen	Asian	Woman	Higher BMI	No
Marie	White	Woman	Mid-Range BMI	Yes
Denise	White	Woman	Higher BMI	Yes
Riley	White	Nonbinary	Mid-Range BMI	Yes
Lily	White	Woman	Mid-Range BMI	No
Brooke	Asian	Woman	Higher BMI	No

a Lower BMI = BMI<18.5, Mid-Range BMI= 18.5-24.9, Higher BMI=BMI>25, determined based on self-reported height and weight.

#### 3.4.1 Theme 1: Community is imperative: “the human connection part of [BAM] is very important” (Marie)

Through semi-structured interviews, it became clear that processing one’s experiences of fatphobia and anti-fat bias during BAM was facilitated by connection with others. Participants valued hearing others’ experiences and ideas and expressed that a sense of community was an essential aspect of BAM that should be retained in a digital adaptation.

##### 3.4.1.1 Subtheme 1: Community alleviates isolation: “I’m not alone in these feelings and these thoughts” (Isabel)

Participants explained that connections were initially formed through a sense of shared experiences. Despite their diverse intersectional identities, participants noted that they struggled in similar ways, which helped them feel less isolated. For example, Alex reflected, “Because I’m an international student, I sometimes think, ‘Well, am I different from others, for example, from a different cultural background?’… Listening to Americans talking about how body size influences their daily life and how they … advocate against anti-fat bias in their interpersonal communications, I … see that, ‘Okay, we’re the same.’ That’s a great encouragement to me that … I’m not alone.” Participants also noted that although they shared overarching struggles with anti-fat bias and fatphobia, these struggles manifested differently for each person. For example, Joey shared, “We tend to only think about our own experience of what it’s like to … have a body we’re in. So it was really interesting to hear … everyone else’s … personal idea of being embodied.” Overall, participants experienced solidarity with others and were able to learn from others’ experiences, which laid the groundwork for collective growth within the group.

##### 3.4.1.2 Subtheme 2: Community empowers change: “discussions really helped me think differently about my own body and society” (Riley)

Ultimately, the sense of community that was established through shared experiences and learning from others allowed participants to meaningfully engage with the intervention and practice combatting fatphobia and anti-fat bias. Every participant identified that interactive exercises (i.e., quick comebacks, role plays), in which participants practiced responding to fatphobic comments or comments exemplifying anti-fat bias, were highly relevant. For example, Isabel shared, “[The quick comebacks and role plays] were important because … when you’re faced with these real-life situations, you don’t always know what the best thing is to say. So, I thought it was really nice that we got … actual situations that could happen. And we got to … work out how to react and respond to those.” Furthermore, Alex shared, “The most shocking and probably effective activities were [the role plays and quick comebacks]. I think that’s a really good practice … in an inclusive environment. Because when I … face those scenarios in my real life, that someone is saying that, ‘You are getting a lot of fat,’ I now got great feedbacks [on how to respond].”

In addition to equipping participants with the confidence to respond to challenging comments exemplifying fatphobia or anti-fat bias in everyday life, the group community also facilitated internal change in participants’ own experiences of fatphobia and anti-fat bias. For example, one BAM activity entails unpacking the associations between weight, health, and worth as a group. Riley expressed that this interactive activity helped them “point out those … little hypocrisies and … why we ascribe … certain aspects of health to worth … which really helped me break down … those attributions to myself.” Furthermore, Helen shared that this activity “increased [her] awareness of [her] underlying bias and unconscious thoughts.” Together as a group, participants developed the skills, confidence, and self-awareness necessary to challenge fatphobia and anti-fat bias both in themselves and in the world around them. Doing these activities in isolation, as expressed by Isabel, would “defeat the whole purpose of the workshop.”

##### 3.4.1.3 Subtheme 3: Community promotes accountability: “[video-based group] meetings would hold me accountable” (Katie)

Participants emphasized that the presence of a community not only enhances the impact of the intervention but also fosters accountability in completing it, particularly when considering a digital adaptation of BAM. For example, Janet shared, “If it’s just … me individually [doing the asynchronous intervention] … it’s just really hard for me to do that on my own.” Similarly, regarding the proposed asynchronous intervention, Isabel expressed, “I’m not sure how motivated I would be to learn these things and do these activities by myself.”

When considering the digital adaptation ideas that have a connection component, participants felt that those that included group-based videoconferencing would promote the greatest amount of accountability, as opposed to connection with a peer facilitator or a group discussion board. Regarding the discussion board, Janet emphasized, “I just don’t think there would be enough accountability for people to post on a discussion board or ask questions … Especially if it’s … an open discussion board, you don’t want to be the first to ask … a sensitive question.” Also regarding the discussion board option, Joey shared, “I just feel like I wouldn’t get anything out of this one … discussion boards never feel connected to me.” Web-based videoconferencing, by contrast, was described as fostering accountability and active engagement. Katie explained that they would “keep me responsible for actually completing the intervention and being able to engage in discussion,” while Marie shared that they would be “really beneficial for accountability.” Overall, participants believed that incorporating synchronous elements like group-based videoconferencing would enhance the digital adaptation’s ability to foster connection and consequently, a sense of accountability.

#### 3.4.2 Theme 2: Flexibility promotes accessibility: “having it digital makes it more accessible” (Marie)

Participants consistently highlighted how the flexibility afforded by a digital adaptation of BAM would significantly enhance accessibility. For those “on a time crunch” (Marcia), the digital format would allow participation without rigid scheduling. Similarly, it would be beneficial for “people who do not feel comfortable joining an in-person group” (Ellen) or those facing logistical barriers such as “transportation, like getting to the facility and back” (Janet). The asynchronous format, in particular, was noted as the most flexible option, enabling individuals to complete the intervention on their own time. Participants also emphasized that this flexibility would facilitate access to the intervention whenever needed. Ellen explained, “I think a lot of people … like the independence and accessibility of an app … assuming that this would be available 24/7. It wouldn’t be like ‘Oh, we have a session at this time, this date.’ Whenever they need to access it, they would have the ability to do so without any restrictions. I think that’s … really important.” Similarly, Katie shared, “[Time] was a barrier for [my participation in BAM] and I had to … move around some things to … get there … so I think definitely having … the time where you can do it asynchronously … would be really helpful and a lot more feasible to … work it into your day.”

#### 3.4.3 Theme 3: Striking a balance with a digital adaptation: “the best of both worlds” (Rachel)

Building on the themes related to community and flexibility, participants emphasized the importance of designing a digital adaptation of BAM that strikes a balance between these elements. They identified the mixed asynchronous and synchronous digital format as a way to preserve the human connection central to BAM while expanding access through flexible, individually-completed components. The majority (n = 8) of participants said that out of all the digital adaptations proposed, the mixed asynchronous and synchronous adaptation is what they would prefer as a replacement for in-person BAM sessions. For example, Marie shared, “I prefer [the mixed synchronous and asynchronous version] because I like the human connection aspect, but then I also think with … a technology-based approach … it makes sense … to have time to … do stuff on your own time.” Furthermore, Riley emphasized that with this hybrid format, as compared to proposed formats without a human interaction component, they would “actually do this work. [They] would actually attend. [They’d] be interested. And [they] wouldn’t … be prompted to … take any shortcuts or try to rush through any work because … it would feel like a more meaningful use of [their] time.” These perspectives reflect participants’ high level of interest in participating in this digital BAM adaptation.

Beyond identifying that a mixed asynchronous and synchronous digital adaptation would preserve the important community aspect of BAM while also promoting greater flexibility, participants offered specific suggestions on how to maximize both elements. For example, to ensure the synchronous component foregrounded connection, participants emphasized smaller groups for videoconferencing. Through small groups, each participant would have an opportunity to contribute to discussion. For example, Riley suggested, “Probably smaller groups of … five to eight … definitely never larger than 10.” Marcia shared, “I’d say … maybe six to seven … eight [people] tops, I think. So everybody can share their ideas.” In general, participants’ recommendations around group size converged between 5 and 10 people. In addition to recommending small group sizes, multiple participants emphasized the importance of establishing norms for participation, such as keeping cameras on and microphones unmuted, to foster a sense of connection and further strengthen the community aspect of the synchronous component.

Participants also shared insights regarding the ideal length and frequency of synchronous sessions. They highlighted that shorter (i.e., less than 2 hours) sessions would be more engaging and easier to integrate into their schedules. For instance, Katie suggested, “I think maybe…45 minutes per session,” while Marcia proposed, “30-minute sessions … or one-hour sessions” as a manageable length for synchronous videoconferencing. Regarding frequency, participants recommended synchronous check-ins at key intervals, such as “at the very beginning, middle, and end” (Marie) of the intervention. Multiple participants suggested dividing the synchronous meetings across specific lessons, such as having “a [group-based videoconference] check-in after the first two lessons and then after the last two” (Riley). Ultimately, shorter sessions with a clear focus that were spread out over time were seen to retain participants’ attention and foster meaningful engagement with a digital adaptation of BAM.

Participants also provided suggestions for the individually completed, asynchronous components. Many emphasized the need for interactivity, whether that be through quizzes, drag-and-drop features, videos, or animations, to “keep it from feeling … like a chore” (Riley). Oftentimes, these suggestions were based on experiences with other apps. For example, Denise recommended, “Anything that seems exciting and is … colorful and … brings us back into it and … keeps that attention span … To use Duolingo as an example … they have the typing, they have the speaking, they have the … putting things in as a puzzle, they have the matching. And so just having different forms of exercises so that it’s not just like, ‘Oh, every time I’m reading the scenario and responding’ or ‘Every time I’m like matching this to that.’” Beyond encouraging interactive components, given their experience completing BAM, participants also had recommendations for specific activities that would be best suited to be completed asynchronously and how to best adapt them. For example, multiple participants expressed that the weight, health, and worth activity could be adapted to an engaging drag-and-drop activity that people could complete on their own then reflect on as a group. Additionally, multiple participants shared that having those completing the intervention identify examples of anti-fat bias and fatphobia on their own and submitting that to facilitators ahead of synchronous sessions would be beneficial.

Ultimately, participants expressed that the synchronous and asynchronous components would nicely complement one another, providing both the flexibility needed to accommodate diverse schedules and the community connection central to BAM’s impact. By combining shorter, focused synchronous sessions with engaging, interactive asynchronous activities, the mixed digital adaptation was seen as “the best of both worlds” (Rachel).

## 4 Discussion

Human-centered design has the potential to improve engagement with and effectiveness of digital interventions for EDs (23). This study informs the development of a digital adaptation of BAM, an ED prevention program that reduces anti-fat bias, fatphobia, and ED psychopathology, by leveraging two phases of human-centered design—investigate and ideate—to explore the experiences of college students with elevated ED psychopathology related to anti-fat bias and fatphobia and to gather feedback from past BAM participants on which aspects of the program should be retained and modified in a digital format. Individuals from both groups also offered feedback on five digital adaptation ideas developed by the study team and BAM peer facilitators. Results offer useful implications for the development of a digital BAM adaptation and future digital mental health research.

First, our findings point to a clear need for interventions that reduce anti-fat bias and fatphobia. All college students with elevated ED psychopathology reported experiences of fatphobia and anti-fat bias that they identified as detrimental to their well-being, aligning with prior research highlighting the pervasive and damaging nature of these experiences (35). The socioecological model of well-being emphasizes the interdependence of intrapersonal, interpersonal, and institutional influences on well-being (36). Thus, the experiences of anti-fat bias and fatphobia that college students reported across domains underscore the importance of addressing these issues at multiple levels through interventions like BAM.

Many college students with ED psychopathology also expressed dissatisfaction with mental health treatment experiences that prevented them from healing their relationships with food and their bodies. One area of dissatisfaction stemmed from a lack of action-oriented support in psychotherapy. This finding mirrors previous research that found that college students desire personal guidance when being treated for mental health concerns (37), demonstrating a need for more structured, action-based mental health interventions for college students. Additionally, multiple participants reported working with mental health providers untrained in EDs, which served as a barrier to overcoming body image and eating concerns. Indeed, there is a documented shortage of providers offering evidence-based treatments for EDs (38). Together, our findings strongly support the need for accessible interventions that integrate actionable strategies to address anti-fat bias, fatphobia, and ED psychopathology—a gap that a digital adaptation of BAM can fill.

Regarding such an adaptation, college students with elevated ED psychopathology and past BAM participants showed similar overall patterns in their ratings of the five proposed digital formats, with average ratings for both groups the lowest for a fully asynchronous adaptation and the highest for a mixed synchronous and asynchronous adaptation. One notable difference was observed with the asynchronous plus peer facilitator format: college students with elevated ED psychopathology rated this option significantly higher than past BAM participants. This preference may reflect participants’ desire for personalized, structured support, particularly given their reported dissatisfaction with unstructured or non-specialized mental health services. In contrast, past BAM participants, who had already experienced the original in-person program and described the essential role of the group community, may have placed comparatively less value on one-on-one facilitator support. Future work should explore how varied levels of facilitator involvement, such as one-on-one support versus group-based interaction, impact engagement, satisfaction, and outcomes among diverse user groups. Overall, though, participants across both groups underscored the importance of (1) social support and (2) flexibility in a digital adaptation of BAM.

Turning first to social support, interaction with others was seen not only as a way to promote engagement and accountability but also as a means of fostering a sense of community. Past BAM participants viewed this sense of community as essential for raising critical consciousness (i.e., recognizing and taking action against systems of oppression) in combatting anti-fat bias and fatphobia. Notably, synchronous group-based videoconferencing was identified as the most effective way to cultivate this community, as real-time interaction fosters an understanding of shared experiences and deeper social connection. These findings align with prior research highlighting the importance of social connection in recovering from body image and eating struggles (39) and in coping with and resisting weight stigma (35).

Although participants emphasized the importance of community-building through synchronous meetings, they also highlighted the value of asynchronous activities in promoting flexibility. These activities would reduce reliance on real-time components while providing accessible support that participants could engage with at their convenience. To ensure asynchronous activities remained engaging, both groups suggested incorporating a variety of interactive features such as quizzes, drag-and-drop exercises, matching activities, and videos, which have previously been identified as facilitators of engagement with digital mental health interventions (40). For example, the BAM activity identifying intrapersonal, interpersonal, and institutional levels of fatphobia and anti-fat bias could be adapted into a drag-and-drop sorting activity, where users classify real-world examples. Additionally, the role play activity could be reimagined as a choose-your-response simulation, allowing users to practice responding to statements rooted in fatphobia or anti-fat bias in a low-stakes manner. For the final reflection activity of sharing one way to act against institutional anti-fat bias, users could submit their commitment to a virtual ‘wall’ and optionally browse anonymized responses from others. While these formats are promising, future research is needed to evaluate their specific impact on reducing anti-fat bias, fatphobia, and ED psychopathology within the context of a digital adaptation of BAM.

The characteristics of the two participant groups provide important context for understanding the qualitative themes that were identified from their feedback on the proposed digital adaptation of BAM. College students with elevated ED psychopathology were more likely to have a lifetime history of an ED and also reported higher fatphobia scores, quantitative findings that align with their identification of fatphobia as a pervasive and detrimental influence. Indeed, prior research demonstrates that fatphobia is a central mechanism of ED onset and maintenance (10, 11). This group also rated the asynchronous plus peer facilitator format significantly higher than past BAM participants, suggesting a desire for structured individualized support, possibly reflecting the severity of their ED symptoms and dissatisfaction with prior treatment. Notably, anti-fat bias scores were comparable across groups, underscoring the robustness of anti-fat bias as a relevant target for BAM. The convergence in qualitative themes—such as the importance of social support, flexibility, and interactivity—despite differences in ED severity and fatphobia, suggests that these preferences may be broadly applicable and valuable to diverse users. Finally, both samples were comprised of young adults, a demographic that has been shown to be open to and benefit from digital mental health interventions (13–18). Given their familiarity with technology and the central role it plays in their daily lives, it is unsurprising that participants emphasized the importance of interactivity and flexibility in a digital adaptation of BAM. Indeed, young adults tend to seek digital experiences that are customizable, engaging, and have gamified elements (13, 19, 21).

Integrating participant feedback into the design of a digital adaptation of BAM is a critical next step. The proposed mixed asynchronous and synchronous format represents a promising starting point, given that both groups rated this format highly, but future research is needed to evaluate its feasibility, acceptability, and effectiveness in reducing anti-fat bias, fatphobia, and ED psychopathology. Additionally, with a digital adaptation, BAM’s reach can be expanded to include populations beyond college students, including individuals outside of college settings or those in larger bodies, and its effectiveness can be tested with these new populations as well.

This study has several notable strengths, one of which lies in its use of human-centered design, which ensures that the digital adaptation of BAM is directly informed by the needs and preferences of its intended users, enhancing its relevance and potential effectiveness. Furthermore, the study addresses a critical gap in the literature by focusing on the integration of anti-fat bias and fatphobia reduction within a digital intervention, an area that has received limited attention despite its importance in ED prevention.

However, limitations of the study must also be acknowledged. Self-selection bias may have influenced the findings, as individuals with a particular interest in addressing anti-fat bias and fatphobia may have been more likely to participate in the study. Similarly, participants’ preference for a format including synchronous components may further reflect selection bias, as those who chose to engage in the initial BAM study—which featured a fully-synchronous, in person format—may be more inclined toward group-based, real-time interaction. However, this limitation can be tempered given that a mixed asynchronous and synchronous format was rated highly across both groups. Additionally, while the study offers initial insights into the acceptability of different digital formats, the findings should be interpreted as preliminary and specific to the participant sample. Future work is needed to explore whether these findings extend to populations with different demographic characteristics and experiences. Furthermore, participant feedback was elicited based on five proposed study formats rather than open-ended, exploratory input, which may have constrained the range of ideas or preferences participants might have otherwise shared. Moreover, the use of digital technology presents its own challenges. Access to and comfort with digital tools, including variation in digital literacy and cultural attitudes toward technology, may affect engagement. Finally, the measure of fatphobia used, the GFFS, has not been validated across genders (Przybyla-Basista et al., 2022) and the measure of anti-fat bias used, the EPSI-NATO, has not been validated across racial and ethnic groups (31). However, the majority of participants identified as White women.

Overall, this study contributes to the growing body of research utilizing human-centered design to develop digital interventions for EDs (22) and addresses the need for digital tools to combat anti-fat bias and fatphobia. The pervasive and harmful experiences of fatphobia and anti-fat bias reported by college students with elevated ED psychopathology, coupled with their struggles to access specific, action-oriented mental health treatment, underscore a significant gap in care that a digital adaptation of BAM could address. Both college students and past BAM participants expressed high interest in a mixed-format digital adaptation that incorporates both synchronous and asynchronous components. To enhance engagement, participants emphasized the importance of synchronous videoconference-based social connection alongside asynchronous interactive features, such as quizzes and matching activities. A critical next step will involve designing and pilot testing this digital adaptation of BAM to evaluate its feasibility and effectiveness.

## Data Availability

The raw data supporting the conclusions of this article will be made available by the authors, without undue reservation.
