# Supplementary material for: Developing a digital intervention to combat fatphobia and anti-fat bias
Source: Front Psychiatry. 2025 May 13;16:1569841. doi: 10.3389/fpsyt.2025.1569841 (PMC12138398; doi:10.3389/fpsyt.2025.1569841)
Supplement: Supplementary file 1 [file Table1.docx]

**Interview for Past BAM Participants**

The following guide reflects the types of questions that will likely be asked of participants during the interview. Some items may change due to the iterative nature of qualitative research. However, the overall topic and level of sensitivity will remain consistent.

**Script**:

START RECORDING

I want to start by saying thank you so much for taking the time to meet with me today – I really appreciate your willingness to take time out of your day to discuss BAM with me! As a reminder, this study seeks to gather your feedback on BAM and any thoughts you have on adapting BAM to a digital format, so that we can hopefully make BAM more accessible and to expand its reach. I have a series of questions to go through with you that should take no more than an hour. After we complete the interview, I will send you a $25 Amazon gift card via email. Any questions before we begin?

Okay, great. To get started, I want to do a little “BAM Refresher” because I know it’s been a few weeks since you completed BAM.

**Show “BAM Refresher” handout**

Here’s an outline of the BAM sessions: so in session 1, we focused on defining fatphobia and anti-fat bias, deconstructing “I feel fat,” and looking at the relationships between weight, health, and worth. In session 2, we debriefed your two homework assignments and completed role plays and quick comebacks so you could practice responding to someone who said something fatphobic or biased against fat people. Any questions about what we did in BAM?

Fantastic! We can get started then. And just a heads up, I will be taking notes during our time today, so if you hear me typing away, that is why.

1. Diving right in, I’d first like to hear your initial thoughts on BAM.
   1. How did you feel about the time commitment?
   2. How relevant did you find BAM to you, personally? Can you give an example?
   3. What information / session / activity stood out to you the most? Why?
   4. What are your thoughts on how similar/different participants were to you in terms of age, gender, race, sexual orientation, etc.? Did this play a role at all in how engaged you were?
      1. How would you feel about being in a group that is more similar or more different to you?
   5. What about for the facilitator? What are your thoughts on how similar/different the facilitator was to you in terms of age, gender, race, sexual orientation, etc.? Did this play a role at all in how engaged you were? Would you rather be with a facilitator who is more similar or more different to you?
2. Now, I’d like to hear about how technology fits into your life. I know technology can mean lots of things – when I say technology during this interview, I will be referring to apps, social media, websites, or any other online resources. To prime you for technology in this context, I have a list of commonly-used mental health technology, which you can see here.

**Show “Mental Health Technology” handout**

- 1. Do you use any technology to talk about or seek support for your mental health or well-being?
     1. If yes:
        1. Tell me a little bit more about your use of [technology referenced].
        2. Why do you use [technology]?
        3. How frequently and when do you use it?
        4. Does it help? Why or why not?
        5. Any specific examples of you using [technology] in your daily life?
        6. **Repeat each set of questions for each different type of technology.**
     2. If no:
        1. Have you ever used any technology to support your mental health or well-being?
           1. If yes:

Could you tell me a bit about what you’ve used?

When did you use these technologies?

Why did you use it?

Did it help? Why or why not?

What did you like about it? Not like about it?

What made you stop using it?

- - - - 1. If no:

Why not?

1. Now, I’d like to dive into any suggestions you have for adapting BAM to a digital format.
   1. First, what ideas do you have for digitizing BAM? Any format that you think would be best? For example, Zoom-based or app-based?
   2. We have five different ideas that I would appreciate your feedback on.

**Show “Digitizing BAM” slides – counterbalance order**

- 1. One idea is to have an intervention that is self-paced and that you would complete individually. What this means is that you would go through the content on your own via a website, app, or other digital platform. It would basically be a space where you could learn about anti-fat bias and fatphobia and reflect on where these things show up in your life. If you had any questions or concerns, you’d be able to reach out to someone trained in the intervention, but you would mostly be on your own.
     1. What are your initial reactions to this idea?
     2. On a scale of 1-5, with 1 being not at all interested and 5 being very interested, how interested would you be in participating in an intervention like this?
  2. One idea is to have the same self-paced, individually-completed intervention, but have the opportunity to connect with a trained peer facilitator over a Zoom call and/or weekly messaging.
     1. What are your initial reactions to this idea?
     2. On a scale of 1-5, with 1 being not at all interested and 5 being very interested, how interested would you be in participating in an intervention like this?
  3. One idea is to have an intervention that you complete on your own time, but that includes a group component where everyone is learning at the same time. So, you would be able to go through the content on your own time throughout a given week, but you could also interact online with others who are going through the same content at the same time. Additionally, you would be able to share your own thoughts and reflections and get support and accountability from the group. Notably, the group would be moderated by someone trained in the intervention, who might encourage discussion by posting interactive questions or additional activities for people to try.
     1. What are your initial reactions to this idea?
     2. On a scale of 1-5, with 1 being not at all interested and 5 being very interested, how interested would you be in participating in an intervention like this?
  4. One idea is to have a format where you would be part of a group that would meet a couple of times on Zoom with a trained facilitator (for example, once at the start and once at the end of the intervention). You would still complete most of the content on your own time, but we would add in a couple of Zoom meetings to help build community within the group, so that you get to know the facilitator and other group members a little bit more during the process and would have the opportunity to share thoughts through verbal discussions.
     1. What are your initial reactions to this idea?
     2. On a scale of 1-5, with 1 being not at all interested and 5 being very interested, how interested would you be in participating in an intervention like this?
     3. When would it be best to have the Zoom meetings?
     4. How many Zoom meetings with the group would you want to have?
     5. How long would you want the Zoom meetings to be?
  5. One idea is to have the intervention be completely synchronous, so you would go through the content and activities with a group and facilitator on Zoom over the course of a few sessions. We would ask you to try out some activities in your life outside of session, but you would not need to complete any additional content for the program other than showing up to each session and participating in the live discussions.
     1. I’ll ask you some questions about number of sessions and how long you’d want them to be in a second, but what are your initial reactions to this idea?
     2. On a scale of 1-5, with 1 being not at all interested and 5 being very interested, how interested would you be in participating in an intervention like this?
     3. How many Zoom meetings with the group would you want to have? How long would you want the Zoom meetings to be?
     4. How many people would you want to be in this group?
  6. Which of these formats would you prefer and why?
  7. Any ideas you have for adapting specific activities to a digital format?
  8. What features would you recommend to make a digital version of BAM more engaging?
  9. Can you think of any challenges that might arise when adapting BAM to a digital format?

1. Is there anything else you’d like to share regarding either your experience with BAM or thoughts on adapting it to a digital format?

Thank you so much for taking the time to meet with me! Your input is so valuable and will help us make BAM more accessible. If anything else comes up, don’t hesitate to reach out. I will be in touch with your payment by the end of the week! :)

STOP RECORDING
