# Supplementary material for: Developing a digital intervention to combat fatphobia and anti-fat bias
Source: Front Psychiatry. 2025 May 13;16:1569841. doi: 10.3389/fpsyt.2025.1569841 (PMC12138398; doi:10.3389/fpsyt.2025.1569841)
Supplement: Supplementary file 2 [file Table2.docx]

**Interview for College Students with Elevated Eating Disorder Pathology**

The following guide reflects the types of questions that will likely be asked of participants during the interview. Some items may change due to the iterative nature of qualitative research. However, the overall topic and level of sensitivity will remain consistent.

**Script**:

START RECORDING

I want to start by saying thank you so much for taking the time to meet with me today – I really appreciate your willingness to take time out of your day to talk about your experiences with me! As a reminder, this study seeks to explore your experiences with body image and eating struggles, as well as any experiences you might’ve had seeking mental health treatment. Some of the questions I ask today may be difficult to think or talk about. I want to emphasize from the get-go that you do not need to answer any questions you do not feel comfortable answering, and if you need a break at any time, just let me know! Any questions before we get started?

1. Okay great! First, I’m curious to hear a little bit more about your struggles with body image and/or food. What does this look like for you right now?
2. Now, I’m curious to hear about your experiences with mental health treatment.
   1. Have you ever been hospitalized, been to a residential treatment center, or been in any intensive treatment setting for mental health concerns?
   2. Have you ever received any mental health treatment or counseling? (clarify outside of higher levels of care if endorsed)
      1. If yes:
         - 1. What was the format (i.e., in person, telehealth, or both; individual or group)? *Note to interviewer: ask the following questions for each format of treatment*

What was this treatment for?

How was that experience for you?

Did you get the kind of help you were looking for? What was helpful? What was not helpful?

- - - - 1. Have you ever wanted [group vs. individual, in-person vs. virtual vs. hybrid] treatment and not gotten it?
    1. If no:
       1. Have you ever wanted treatment or counseling and not gotten it?
          1. If yes: What kept you from getting mental health treatment or counseling?

1. Thank you for sharing. Now, I’d like to get a sense of your knowledge of the terms “anti-fat bias” and “fatphobia.” These terms are not super well-known, so no worries if you’re not sure exactly what they mean!
   1. When I say “anti-fat bias,” what comes to mind? How would you define this term?
   2. When I say “fatphobia,” what comes to mind? How would you define this term?

For the study today, we’ll think of fatphobia as the fear of weight gain present *within* individuals and anti-fat bias as negative evaluations *towards another person* based on their weight, specifically if a person is not thin and/or muscular. So fatphobia refers an individual’s own fears related to weight gain and fatness – related to themselves, and anti-fat bias refers to bias or stigma related to others’ fatness.

Any questions about those definitions?

1. How does fatphobia (i.e., a fear of being fat) show up in your struggles with body image and food?
   1. How have these experiences affected your mental health or well-being?
   2. What instances or contexts have you noticed this fatphobia?
2. Have you ever noticed fatphobia in others, such as friends or family members exhibiting fear of being fat?
   1. If yes: How have these experiences affected your mental health or well-being?
   2. If yes: What instances or contexts have you noticed fatphobia in others?
3. This one can be hard to talk about, but have you ever noticed anti-fat bias in yourself? For example, assuming a fat friend wouldn’t be interested in doing active outings, or assuming that a fat peer isn’t smart or would produce sloppy work. Again, I know these things can be hard to admit and talk about, but thoughts like these are very common (I’ve unfortunately even had similar thoughts myself at times) so if there’s anything you can think of, I’d be interested to hear what you have to share.
   1. If yes: What instances or contexts have you noticed this anti-fat bias popping up within yourself?
   2. If yes: How have these experiences affected your mental health or well-being?
4. Have you ever been the one on the receiving end of anti-fat bias? For example, have others made assumptions about your physical health or work ethic based on your body size? Or have you been teased for being fat? Again, I know this can be hard to talk about, but I appreciate your willingness to share.
   1. If yes: How have these experiences affected your mental health or well-being?
   2. If yes: Are there particular instances or contexts where you’ve noticed this anti-fat bias more prominently?
   3. If yes: How did you react to this? For example, did you brush it off, not think much of it, or try to change it?
5. What about in your community—among family members, friends, peers? Have you ever noticed others *directly experiencing* anti-fat bias? For example, have you ever noticed others being teased? Or have others received negative comments from healthcare providers?
   1. If yes: How have these experiences affected your mental health or well-being?
   2. If yes: What instances or contexts have you noticed this anti-fat bias more prominently?
   3. If yes: How did you react to this? For example, did you brush it off, not think much of it, or try to change it?
6. What about others demonstrating anti-fat bias?
   1. If yes: How have these experiences affected your mental health or well-being?
   2. If yes: What instances or contexts have you noticed this anti-fat bias more prominently?
7. Now, I’d like to hear about how technology fits into your life. I know technology can mean lots of things – when I say technology during this interview, I will be referring to apps, social media, websites, or any other online resources. To prime you for technology in this context, I have a list of commonly-used mental health technology, which you can see here.

**Show “Mental Health Technology” handout**

- 1. Do you use any technology to talk about or seek support for your mental health or well-being?
     1. If yes:
        1. Tell me a little bit more about your use of [technology referenced].
        2. Why do you use [technology]?
        3. How frequently and when do you use it?
        4. Does it help? Why or why not?
        5. Any specific examples of you using [technology] in your daily life?
        6. **Repeat each set of questions for each different type of technology.**
     2. If no:
        1. Have you ever used any technology to support your mental health or well-being?
           1. If yes:

Could you tell me a bit about what you’ve used?

When did you use these technologies?

Why did you use it?

Did it help? Why or why not?

What did you like about it? Not like about it?

What made you stop using it?

- - - - 1. If no:

Why not?

1. Thanks so much for answering all my questions so far, we just have one more section of the interview! My team and I are in the process of developing a digital intervention that addresses anti-fat bias and fatphobia and would love to hear your thoughts on how this intervention might take shape. We have five different ideas that I would appreciate your feedback on.

**Show “Digitizing BAM” slides – counterbalance order**

- 1. One idea is to have an intervention that is self-paced and that you would complete individually. What this means is that you would go through the content on your own via a website, app, or other digital platform. It would basically be a space where you could learn about anti-fat bias and fatphobia and reflect on where these things show up in your life. If you had any questions or concerns, you’d be able to reach out to someone trained in the intervention, but you would mostly be on your own.
     1. What are your initial reactions to this idea?
     2. On a scale of 1-5, with 1 being not at all interested and 5 being very interested, how interested would you be in participating in an intervention like this?
  2. One idea is to have the same self-paced, individually-completed intervention, but have the opportunity to connect with a trained peer facilitator over a Zoom call and/or weekly messaging.
     1. What are your initial reactions to this idea?
     2. On a scale of 1-5, with 1 being not at all interested and 5 being very interested, how interested would you be in participating in an intervention like this?
  3. One idea is to have an intervention that you complete on your own time, but that includes a group component where everyone is learning at the same time. So, you would be able to go through the content on your own time throughout a given week, but you could also interact online with others who are going through the same content at the same time. Additionally, you would be able to share your own thoughts and reflections and get support and accountability from the group. Notably, the group would be moderated by someone trained in the intervention, who might encourage discussion by posting interactive questions or additional activities for people to try.
     1. What are your initial reactions to this idea?
     2. On a scale of 1-5, with 1 being not at all interested and 5 being very interested, how interested would you be in participating in an intervention like this?
  4. One idea is to have a format where you would be part of a group that would meet a couple of times on Zoom with a trained facilitator (for example, once at the start and once at the end of the intervention). You would still complete most of the content on your own time, but we would add in a couple of Zoom meetings to help build community within the group, so that you get to know the facilitator and other group members a little bit more during the process and would have the opportunity to share thoughts through verbal discussions.
     1. What are your initial reactions to this idea?
     2. On a scale of 1-5, with 1 being not at all interested and 5 being very interested, how interested would you be in participating in an intervention like this?
     3. When would it be best to have the Zoom meetings?
     4. How many Zoom meetings with the group would you want to have?
     5. How long would you want the Zoom meetings to be?
     6. How many people would you want to be in this group?
  5. One idea is to have the intervention be completely synchronous, so you would go through the content and activities with a group and facilitator on Zoom over the course of a few sessions. We would ask you to try out some activities in your life outside of session, but you would not need to complete any additional content for the program other than showing up to each session and participating in the live discussions.
     1. I’ll ask you some questions about number of sessions and how long you’d want them to be in a second, but what are your initial reactions to this idea?
     2. On a scale of 1-5, with 1 being not at all interested and 5 being very interested, how interested would you be in participating in an intervention like this?
     3. How many Zoom meetings with the group would you want to have? How long would you want the Zoom meetings to be?
     4. How many people would you want to be in this group?
  6. Based on these ideas, which would you prefer? Do you have any other ideas or suggestions for digital platforms we could use for the intervention?
  7. Would you want the other participants in your group to be more similar to you in terms of age, gender, race, sexual orientation, etc., or more different? What about the facilitator?

1. Any other thoughts or feedback based on what we’ve discussed today?

What is your address?

That’s all I have! Thanks so much for your time and input, I really appreciate it and will be in touch with your payment by the end of the week! :)

STOP RECORDING
